# Determination of Ochratoxin A contamination in grapes, processed grape products and animal-derived products using ultra-performance liquid chromatography-tandem mass spectroscopy system

**DOI:** 10.1038/s41598-018-20534-7

**Published:** 2018-02-01

**Authors:** Dongmei Wei, Xiaohu Wu, Jun Xu, Fengshou Dong, Xingang Liu, Yongquan Zheng, Mingshan Ji

**Affiliations:** 1 College of Plant Protection, Shenyang Agricultural University, Shenyang, 110161 China; 2 Risk Assessment Laboratory for biological hazards of agricultural product quality and safety, Ministry of Agriculture, Institute of Plant Protection, Chinese Academy of Agricultural Sciences, Beijing, 100193 China

## Abstract

We developed a sensitive and rapid analytical method to determine the level of Ochratoxin A contamination in grapes, processed grape products and in foods of animal origin (a total of 11 different food matrices). A pretreatment that followed a “quick, easy, cheap, effective, rugged, and safe” protocol was optimized to extract Ochratoxin A from the matrices, and the extracted Ochratoxin A was then detected with the use of a highly sensitive ultra-performance liquid chromatography-tandem mass spectrometry system. Good linearities of Ochratoxin A were obtained in the range of 0.1–500 µg L^−1^ (correlation coefficient (R^2^) > 0.9994 in each case). Mean recovery from the 11 matrices ranged from 70.3 to 114.7%, with a relative standard deviation ≤19.2%. The method is easy to use and yields reliable results for routine determination of Ochratoxin A in food products of grape and animal origin. In store-purchased foods and foods obtained from the field and wholesale suppliers, the Ochratoxin A concentration ranged from undetectable to 10.14 µg kg^−1^, with the more contaminated samples being mainly those of processed grape products. Our results indicate that the necessity for regulation of and supervision during the processing of grape products.

## Introduction

Ochratoxin A (OTA) is a secondary fungal metabolite produced mainly by *Aspergillus* and *Penicillium* species^1,2^. OTA exhibits potent nephrotoxicity, hepatotoxicity, cytotoxicity, and immunotoxicity and is a potential carcinogen^3–5^. OTA has been classified as a possible human carcinogen (group 2B) by the International Agency for Research on Cancer^6^. OTA may reach the human food chain in contaminated, mostly cereal-based feed ingested by animals that are fated to become food for human consumption^7,8^. Brera and colleagues^9^, Endou and colleagues^10^, and Krogh^11^ found that OTA contamination in foods correlates with endemic nephropathy in humans and pigs. OTA is found predominantly in grain, animal feed, fruit^12^, and processed foods of varying origin, with a wide range and low concentrations. The European Union has set a maximum concentration for OTA in plant-derived products, including cereal, coffee, and grape-based foods, as between 0.5 to 10 µg kg^−1 ^^13^, but for animal-derived food no recommendation concerning a maximum level has been made. To better understand the extent to which agricultural products are contaminated with OTA and allow for risk assessment, a highly sensitive and reliable analytical method is needed to determine OTA levels in agricultural products, especially animal-derived products.

Extraction of target analyte from matrix and subsequent quantification are the two key steps for analysis of a contaminating compound. Most reports concerning OTA detection have focused mainly on contamination of cereals, coffee^14^, fruits^15–18^, and pork products^19–21^. These methods mainly have used a pretreatment step that relies on immunoaffinity column and analysis by HPLC-FLD. However, because the methods involves blending, quantitative transfers, wash the immunoaffinity column, elute the target compound, and evaporate and exchange the solvent, which are procedures that are complicated, time-consuming, and require large amounts of solvents. Also, each step is a potential source of systematic and random errors. In addition, since the average levels of OTA are lower in agricultural products, the detectability and sensitivity of analytical methods used should be very high, in the reported method, the limit of quantitation (LOQ) for all sample type were between 0.2 to 1 µg kg^−1 ^^14,15,20,21^, it may higher than the OTA levels in real samples. Hence, development of a simple and sensitive pretreatment would be very useful.

QuEChERS (Quick, Easy, Cheap, Effective, Rugged, and Safe) methods usually use acetonitrile (MeCN) partitioning and a dispersive solid-phase extraction (d-SPE) clean-up^22^. Because of their simplicity, high efficiency, and cost-effectiveness, QuEChERS procedures have been applied to the analysis of mycotoxins in barley^23^, rice^24^, and animal feed^25^. Furthermore, QuEChERS methods are flexible, as the optimization of their pretreatment processes allows for the extraction of target compounds from complex matrices including foods of animal origin^26^. In addition, an ultra-performance liquid chromatography (UPLC) system coupled to a Xevo TQ-S triple quadrupole mass spectrometer serves as a powerful tool for the determination of trace levels of pesticides^27^ and mycotoxins^28^ from various matrices. The Xevo TQ-S^29^ is equipped with patented StepWave ion-guide technology, which improves ion sampling in the source and ion-transfer efficiency, resulting in excellent sensitivity. Therefore, we posited that the combined use of a QuEChERS pretreatment with a UPLC-Xevo TQ-S system should allow for quantification of trace levels of OTA in foods of plant and animal origins.

The aim of this study was thus to establish a rapid and sensitive analytical method to determine the extent to which OTA residue contaminates grapes, grape-related matrices (grape wine, grape juice, and raisins), and foods of animal origin (pork, porcine kidney, liver, and fat; chicken and eggs; cow milk). To maximize purification and recovery of OTA in the matrices, the ability of MeCN containing varying amounts of acetic acid (HOAc) to quantitatively extract OTA was assessed. To our knowledge, this is the first time a QuEChERS method and UPLC-tandem mass spectroscopy have been employed to quantify OTA levels in foods of animal origin. Furthermore, we collected 717 food samples from local markets, fields, and wholesale supply warehouses and analyzed them for their OTA content to make risk assessments concerning OTA contamination in foods available for human consumption.

## Results

### Linearity and LOQ

The optimized method was used to quantify OTA spiked into the various matrices. Calibration curves at concentration between 0.1 µg L^−1^ and 500 µg L^−1^, each using the results for six solutions spiked with the standard solution of OTA, were prepared for each matrix. The linear regression equations, and R^2^ values for the matrix-matched curves are given in Table 1. For each curve, an R^2^ value ≥ 0.9994 was obtained, and the LOQ was 0.1 µg kg^−1^.

**Table 1:** Calibration equations, R^2^ values, LOQ values, and matrix effects for Ochratoxin A detection by our optimized UPLC-MS/MS method.

Matrix	Regression equation	R^2^	Slop ratio^a^	Matrix effect^b^ (%)	LOQ(µg kg^−1^)
Acetonitrile	y = 3417.5x − 7266.9	R² = 0.9999			0.1
Grape	y = 4558.9x + 800.35	R² = 1	1.33	33	0.1
Wine	y = 5406.2x + 8354.5	R² = 0.9997	1.58	58	0.1
Juice	y = 6261.2x + 10692	R² = 0.9997	1.83	83	0.1
Raisin	y = 6011.2x − 2406.7	R² = 1	1.76	76	0.1
Pork	y = 4309.4x + 1248.5	R² = 1	1.26	26	0.1
Porcine liver	y = 3206.1x − 3857.9	R² = 0.9999	0.94	−6	0.1
Porcine Kidney	y = 5251.4x + 4169.7	R² = 1	1.54	54	0.1
Porcine fat	y = 5673.7x + 8237.3	R² = 0.9999	1.66	66	0.1
Chicken	y = 4910.9x − 740.04	R² = 0.9999	1.44	44	0.1
Eggs	y = 4723.7x + 13969	R² = 0.9994	1.38	38	0.1
Milk	y = 5459.1x + 10234	R² = 0.9998	1.60	60	0.1

### Matrix effects

Matrix effects are a major challenge when attempting to quantify a contaminant by LC-MS/MS because co-eluting sample constituents may enhance or suppress the analyte response, which is also dependent on the instrument, the properties of the analyte and its concentration, the matrix, and the sample pretreatment procedure^30^. We assessed the effect of each matrix on MS/MS detection of OTA, the ratio of the slope of each of the curves for spiked OTA samples from each matrix to that of the standard solvent curve and the values of each matrix effect are shown in Table 1. Enhancement or suppression (from −6 to 83%) of the OTA signal was observed for the 11 matrices. An OTA signal enhancement effect (26% ≤matrix effect ≤83%) was observed for all matrices except for porcine liver, for which a signal suppression effect was evident (matrix effect = −6%). Therefore, matrix-matched calibration standards were necessary to accurately quantify OTA extracted from the different matrices.

### Precision and accuracy

The precision and accuracy of the method were evaluated according to the recoveries obtained for blank samples analyzed in quintuplicate and spiked with OTA at 0.1, 1, 10, or and 500 µg kg^−1^. Mean recovery ranged from 70.3 to 114.7% with RSD values <19.2% for all samples (Table 2); the RSD_r_ values ranged from 0.8 to 19.2%, and the RSD_R_ values ranged from 2.0 to 16.9%_._ These results indicate that the method achieved satisfactory recovery, precision, and sensitivity values for the detection of OTA in all tested matrices.

**Table 2:** Accuracy and precision of the optimized method for the 11 matrices, each of which was spiked with one of four concentrations of Ochratoxin A.

Matrix	Spiked level (µg kg^−1^)	Intra-day (n = 5)						Inter-day(n = 15) RSD(%)
		Day 1		Day 2		Day 3		
		Average recoveries(%)	RSDr(%)	Average recoveries(%)	RSDr(%)	Average recoveries(%)	RSDr(%)	
Grape	0.1	71.7	5.4	80.1	3.4	80.0	10.3	8.2
	1	110.2	2.5	109.4	3.4	107.5	2.5	2.8
	10	110.7	1.4	110.3	1.3	103.1	4.2	4.1
	500	114.7	1.1	102.6	2.6	100.1	0.8	6.4
Wine	0.1	80.0	5.8	80.6	4.2	85.8	2.4	5.0
	1	96.5	6.0	94.7	10.0	98.7	2.6	6.5
	10	93.4	3.0	91.3	4.1	98.6	1.7	4.4
	500	111.2	0.8	109.9	1.5	96.0	8.7	8.0
Juice	0.1	79.7	6.6	79.2	5.2	82.5	10.8	7.2
	1	95.7	4.3	108.5	1.3	93.1	1.8	4.3
	10	93.4	3.0	111.7	1.8	101.9	3.7	6.8
	500	110.6	2.1	94.7	10.0	109.0	1.3	2.0
Raisin	0.1	80.7	3.9	85.9	8.1	74.3	6.6	8.4
	1	106.9	3.0	100.2	5.1	82.9	7.2	11.8
	10	87.0	7.4	99.5	4.4	94.6	4.6	7.6
	500	109.0	2.0	102.6	6.4	96.5	4.2	6.6
Pork	0.1	90.2	3.0	79.9	8.6	83.0	9.5	8.4
	1	97.5	7.1	102.5	3.2	89.4	2.6	7.3
	10	74.9	2.3	80.4	10.8	94.6	3.6	12.0
	500	75.1	9.8	78.4	11.6	99.3	2.3	15.2
Porcine liver	0.1	76.8	3.5	73.9	5.7	77.9	12.2	7.4
	1	84.4	4.2	103.5	4.6	100.1	6.2	10.1
	10	84.3	9.8	104.6	6.3	100.4	3.8	11.3
	500	93.3	4.7	97.7	3.3	96.0	3.4	4.1
Porcine kidney	0.1	84.4	9.4	87.5	17.2	87.7	10.4	11.3
	1	77.4	6.7	74.4	3.9	93.4	6.8	12.0
	10	102.9	16.4	81.8	15.3	91.3	5.7	15.8
	500	87.3	6.3	91.2	9.5	98.8	5.0	8.5
Porcine fat	0.1	79.9	8.3	71.6	4.1	77.8	10.4	8.6
	1	73.0	1.7	90.5	15.1	71.4	5.4	15.0
	10	75.7	3.6	70.7	8.4	74.2	4.1	6.0
	500	70.3	3.6	103.3	7.1	92.1	5.2	16.9
Chicken	0.1	81.1	12.5	85.6	14.6	80.5	7.7	11.2
	1	108.8	7.8	88.2	3.7	83.9	4.4	13.1
	10	73.8	9.0	83.6	19.2	72.5	2.0	13.8
	500	73.7	8.5	75.6	8.9	78.8	8.9	8.6
Eggs	0.1	75.5	12.4	80.5	4.6	81.0	5.1	7.9
	1	97.1	4.8	101.1	7.6	99.4	4.5	5.6
	10	83.2	11.5	93.8	11.9	95.8	16.7	14.2
	500	87.0	3.0	80.4	3.5	86.9	3.3	4.8
Milk	0.1	84.0	5.8	90.9	4.3	96.9	3.9	7.4
	1	94.8	13.9	90.4	12.0	84.1	7.5	12.0
	10	86.1	6.4	100.6	2.0	104.1	12.6	9.0
	500	89.9	14.8	89.9	6.6	93.8	8.0	4.2

### Occurrence of OTA in real samples

The ability of the method to measure trace levels of OTA was evaluated by analyzing 717 samples obtained from fields, wholesale warehouses, and stores. OTA was found at concentrations of 0.35, 0.61, 1.11 µg kg^−1^ in three grape samples acquired from a field in Xinjiang province. For three raisin samples from a market in Tianjin, the concentration of OTA was 10.14 µg kg^−1^, which exceeds the maximum allowed concentration for OTA in raisins as established by the EU (10 μg kg^−1^)^13^, whereas the other two concentrations were 0.18 and 0.43 μg kg^−1^. For 6 of the 41 grape juice samples, the detected OTA concentration was between 0.1 and 0.2 μg kg^−1^, in other 35 grape juice samples, OTA could be detected but the concentration was below the LOQ, whereas for the remaining samples, OTA could not be detected. Information concerning the occurrence of OTA in real samples is shown in Table 3. The high occurrence of OTA in the grape juice and unacceptably high contamination level of one raisin sample indicate that supervision of the farm-to-store chain for grape juice and raisins is necessary, as is quantification of OTA in those foods.

**Table 3:** Occurrence of Ochratoxin A in samples obtained in the field.

Sample type	No. of samples analyzed	No. of Positive samples	Incidence of positives (%)	Minimum (µg/kg)	Maximum (µg/kg)
Grape	320	3	0.93	0.35	1.11
Wine	21	0	0	ND	ND
Juice	41	41	100	<LOQ	0.17
Raisin	195	3	1.54	0.18	10.14
Pork	20	0	0	ND	ND
Porcine liver	20	0	0	ND	ND
Porcine kidney	20	0	0	ND	ND
Porcine fat	20	0	0	ND	ND
Chicken	20	0	0	ND	ND
Eggs	20	0	0	ND	ND
Milk	20	0	0	ND	ND

## Discussion

To select the multiple reaction-monitoring parameters, the standard solution of OTA (100 µg L^−1^) was directly injected into the UPLC-ESI-MS mass spectrum at different cone voltage. Excellent sensitivity was achieved in the negative ionization mode, the deprotonated molecule at m/z = 402.11 was selected as precursor MS ion, the final cone voltage was 34 V. The precursor ion was fragmented at different collision energies, and the selected reaction-monitoring parameters were optimized to obtain the highest degree of sensitivity. The two most intense fragment ions were observed in the product-ion spectra—one at *m/z* = 167.01 and the second at *m/z* = 211.02; these two ions were used for quantitative and qualitative analysis of the OTA content in the samples when the collision energy was 36 V or 26 V, respectively.

**Figure 1:**
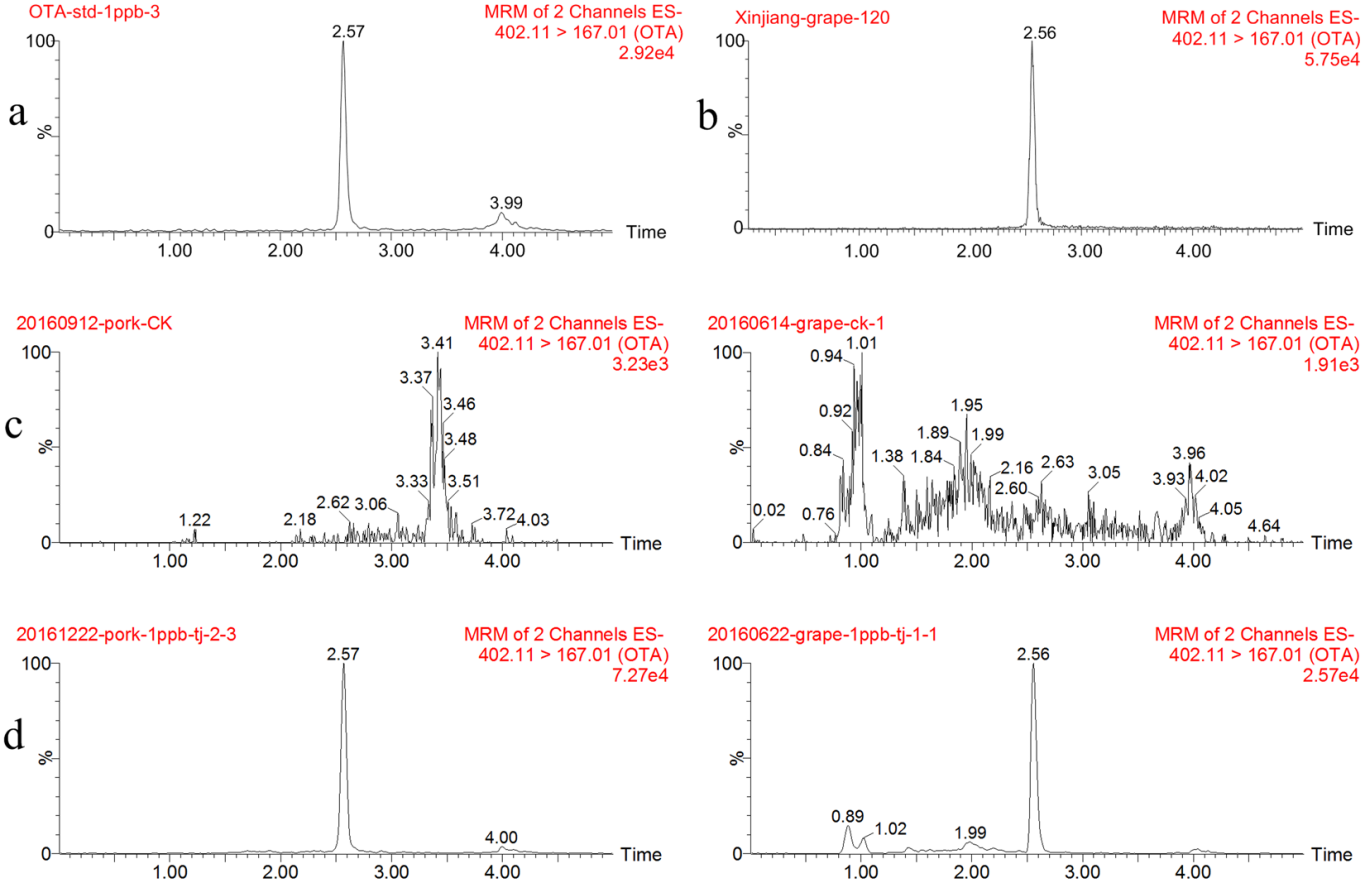
Typical UPLC-MS/MS multiple reaction-monitoring chromatograms for Ochratoxin A (**a**) in a standard solution (1 μg L^−1^), (**b**) from a grape sample collected at a field in Xinjiang province, (**c**) from unspiked pork and grape samples, and (**d**) from pork and grape samples each spiked at 1 μg L^−1^.

To optimize the peak shapes and retention behavior, different mobile-phase compositions MeCN and water, MeCN and 0.2% (v/v) formic acid_(aq)_, methanol and water, and methanol and 0.2% (v/v) formic acid_(aq)_ were assessed. The methanol/0.2% formic acid_(aq)_ produced the best combination of satisfactory retention time (2.57 min) and peak shape for OTA with no interfering peaks (Fig. 1).

**Figure 2:**
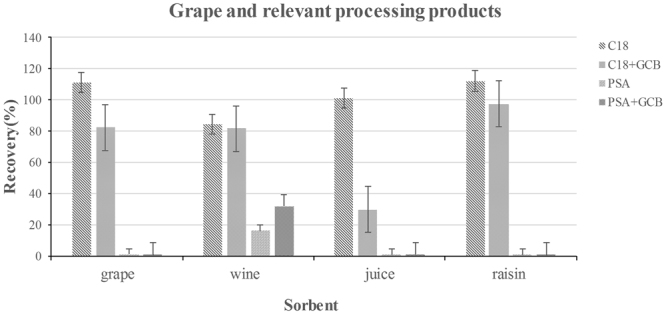
Effect of different types of sorbents on the detection of spiked Ochratoxin A (10 µg kg^−1^) in different processed grape products (n = 3 per matrix).

**Figure 3:**
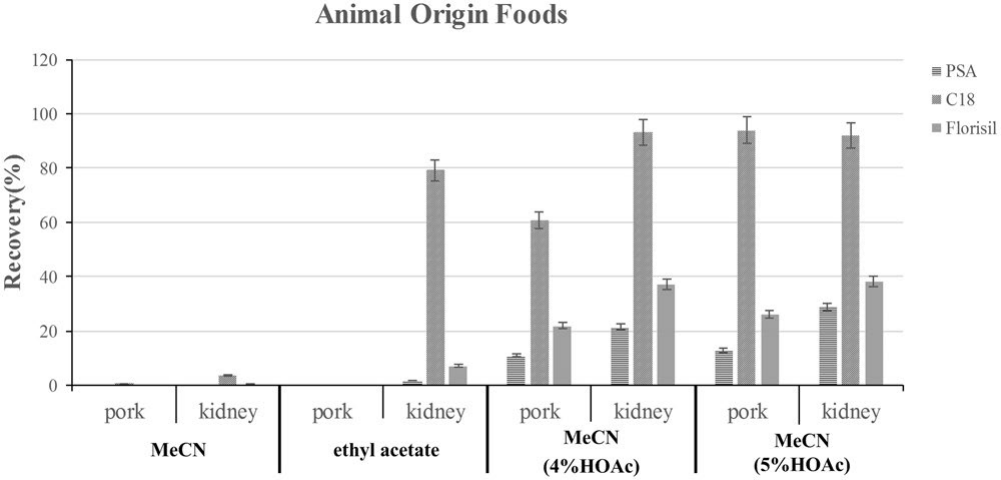
Effect of different extraction solvents and sorbents on the detection of spiked Ochratoxin A (10 µg kg^−1^) in different foods of animal origin (n = 3 per matrix).

The selection of an extraction solvent and sorbent is critical for the detection of a target compound in different matrices. We first considered MeCN as the extraction solvent because its use usually results in better recoveries with less co-extraction of matrix components^31^. D-SPE is a clean-up technique that involves mixing the sample extract with a mixture of anhydrous MgSO_4_ and sorbents to remove matrix components. Common sorbents include primary secondary amine (PSA), octadecylsilane (C18), graphitized carbon black (GCB), and Florisil, which remove different types of impurities based on chemical properties. PSA functions as a weak anion exchanger and has a strong sorption capacity for free fatty acids and other polar pigments, whereas C18 is a common reverse-phase sorbent and is suitable for extraction of nonpolar and partially polar compounds from polar samples. GCB is mainly used to remove pigments, and Florisil is used to separate and remove matrix secretions and grease. We assessed the ability of the following sorbents to remove contaminants from the grape and processed grape samples: 50 mg PSA; 50 mg PSA + 10 mg GCB; 50 mg C18; and 50 mg C18 + 10 mg GCB. We also assessed the following sorbents to remove contaminants from foods of animal origin: 50 mg PSA; 50 mg C18; and 50 mg Florisil. Pure MeCN resulted in satisfactory OTA recovery for the grape, grape wine, grape juice, and raisin samples when C18 was the sorbent (recoveries were between 84.56% and 112.05%; Fig. 2). However, extraction of OTA from foods of animal origin was not satisfactory when pure MeCN served as the extraction solvent (recoveries were <3.72%; Fig. 3). Then we assessed the ability of ethyl acetate to extract OTA from pork meat and porcine kidney, OTA recovery from porcine kidney was satisfactory when C18 was the sorbent (79.30%), but ethyl acetate could barely extract OTA from pork meat. Because organic solvents mixed with acidic solutions are often used to extract OTA from solid samples^32^, we assessed the ability of MeCN containing 4 or 5% (v/v) HOAc to extract OTA from pork meat and porcine kidney **(**Fig. 3). Recoveries increased as the concentration of HOAc increased when C18 was the sorbent. OTA recovery from pork was not satisfactory with use of MeCN containing 4% HOAc (recovery 55.03–69.04%); when HOAc was increased to 5%, however, mean recovery increased substantially (93.98% and 92.01% for the pork and kidney samples, respectively). The efficiency of this combination of extraction solution and sorbent was acceptable for all foods of animal origin. Given these results, we used MeCN as the extraction solvent for grapes and processed grape products, and MeCN containing 5% HOAc (v/v) was used for all foods of animal origin. As shown in Figs 2 and 3, Recovery and RSD values were always acceptable when 50 mg of C18 was used to purify OTA from a matrix (Figs 2 and 3). Therefore, C18 was used as the sorbent for all additional experiments.

## Conclusions

In the present study, we established and validated a simple and robust analytical method using UPLC-MS/MS in the negative ionization mode to detect OTA in grapes, processed grape products, and foods of animal origin. OTA eluted within 5 min with satisfactory recovery, linearity, accuracy, precision and a low LOQ. The detection of OTA in real samples using the method confirmed its reliability and efficacy for the routine monitoring of OTA in grapes, processed grape products, and foods of animal origin. Identification of OTA in samples from the food chain revealed that OTA may be present at high levels in raisins, mostly, at low levels in grapes and grape juice, although in one raisin sample the amount of OTA was above the maximum allowable limit, suggesting that supervision during grape product processing will be needed.

## Materials and Methods

### Chemicals and reagents

OTA (99.76%, analytical standard grade) was obtained from Fermentek Ltd. HPLC-grade methanol was purchased from Sigma-Aldrich (Steinheim, Germany). Anhydrous MgSO_4_ (analytical grade) NaCl, MeCN, and HOAc were purchased from Beihua Fine-Chemicals Co. (Beijing, China). Ultrapure water was prepared using a Milli-Q system (Bedford, MA, USA). PSA (40 µm), C18 (40 µm), Florisil, and GCB (40 µm) sorbents and 0.22-µm pore nylon syringe filters were provided by Agela Technologies Inc. (Tianjin, China).

A standard stock solution (100 mg L^−1^) of OTA was prepared in MeCN. Standard working OTA solutions at concentrations of 0.1, 1, 5, 10, 100 and 500 µg L^−1^ were prepared by diluting samples of the standard stock solution with MeCN. Accordingly, matrix-matched standard solutions of 0.1, 1, 5, 10, 100 and 500 µg L^−1^ OTA were obtained by adding an appropriate volume of a sample extract (grapes, grape wine, grape juice, raisins, pork, porcine kidney, liver, or fat, and chicken, eggs, or milk) to each serially diluted standard solution. All solutions were protected against light and stored in a refrigerator at −20 °C until used.

### UPLC-MS/MS analysis

A Waters ACQUITY UPLC system (Milford, MA, USA) consisting of a UPLC binary solvent manager, a UPLC manager, and a column heater equipped with a UPLC HSS T3 column (2.1 × 100 mm, 1.8-μm particle size) was used. The mobile phase consisted of methanol (solvent A) and 0.2% (v/v) formic acid_(aq)_ (solvent B). Elution was performed in the gradient mode (0 min, 10% A; 1.5 min, 90% A; 3.0 min, 90% A; 3.1 min, 10% A; 5.0 min, 10% A) for a total of 5 min. The flow rate was 300 µL/min, and the injection volume was 5 µL. Temperatures of the sample manager and column were 5 and 40 °C, respectively.

Qualitative and quantitative analyses of OTA were performed using a Waters XEVO TQ-S tandem quadrupole mass spectrometer equipped with an electrospray ionization source. Nitrogen (99.95%) and argon (99.999%) each at a pressure of 2 × 10^–3^ mbar served as the nebulizer and collision gas in the T-wave cell, respectively. Tandem mass spectral detection was performed by multiple reaction monitoring in the negative ionization mode. The monitoring conditions were optimized for OTA. All parameters for the multiple reaction-monitoring transitions, cone voltage, and collision energy were optimized to obtain the best sensitivity and resolution. Typical parameters were: capillary voltage, 3.1 kV; source temperature, 150 °C; desolvation temperature, 200 °C. Nitrogen was the cone and desolvation gas supplied at 150 and 800 L h^−1^, respectively. MassLynx software (version 4.1) was used to collect and analyze the data.

### Field sample collection and preparation

A total of 717 samples were collected from the main production areas and/or the major provinces of China in 2016 (Table 4). The grape samples were collected from a field located in each county of the main production areas at harvest. Milk samples were collected from cows that had been pastured in the main production areas. Samples of processed grape products and foods of animal origin were collected from wholesaler supply warehouses or at large supermarkets in the major provinces and municipalities in China. For each collection, product was sampled throughout the location. Sample sizes were a minimum of 1 kg. Samples were sent to the laboratory immediately after acquisition where they were chopped, homogenized, and stored at −20 °C until used.

**Table 4:** Geographical locations and numbers of the samples acquired in different provinces of China.

	Grape	Wine	Juice	Raisin	Pork	Porcine liver	Porcine Kidney	Porcine fat	Chicken	Eggs	Milk
Shanxi	27	5	10	2							
Ningxia	32	1	5	3							7
Gansu	35	5									
Xinjiang	53	4		129							7
Hebei	45		21	20	3	3	3	3			
Shandong	35	4		5	3	3	3	3			
Beijing	15								4	3	
Yunnan					2	2	2	2			
Anhui					1		2	2			
Hubei							2	2		1	
Jiangsu									1		
Hainan									4		
Zhejiang										5	
Tianjin	40	2	5	36					7	7	
Jilin	38										
Henan					4	4	4	4			
Sichuan					4	4	4	4			
Guangdong					3	4			4	4	
Inner Mongolia											6
total	320	21	41	195	20	20	20	20	20	20	20

Each thoroughly homogenized 10-g matrix sample was added into a 50-mL Teflon centrifuge tube. Next, 10 mL of MeCN was added into the tubes containing grape, grape wine, grape juice, or raisins, and 10 mL MeCN containing 5% (v/v) HOAc_(aq)_ was added into the tubes containing pork, porcine kidney, liver, or fat, chicken, eggs, or milk. The tubes were shaken vigorously for 10 min. Next, 4 g of anhydrous MgSO_4_ and 1 g of NaCl were added, and the tubes were vortexed again for 5 min. Samples were then centrifuged at 2811 × *g* for 5 min. Next, the upper 1.5-mL volume of each sample was transferred into a 2.0-mL centrifuge tube containing 50 mg of C18 sorbent and 150 mg of anhydrous MgSO_4_. Then, the tubes were vortexed for 1 min and centrifuged for 5 min at 2400 × *g*. Each upper layer was filtered through a new 0.22-µm nylon syringe filter and then transferred into a brown sampler vial for UPLC-MS/MS.

### Method validation

The method was validated in terms of its sensitivity, accuracy, precision, specificity, and linearity. The limit of quantification (LOQ), a measure of a method’s sensitivity, was established based on the lowest spiked concentration in each matrix that had a recovery value between 70 and 120% and a relative standard deviation (RSD) ≤20%^33^.

The assays used to determine recovery in terms of precision and accuracy were performed using spiked OTA samples at a concentration of 0. 1, 1, 10, or 500 µg kg^−1^. Five replicates of each spiked sample at a given OTA concentration were prepared by adding the appropriate volume of the standard solution to a matrix sample, allowing the samples to settle for 30 min, and then extracting and purifying the OTA in the samples according to the associated sample-preparation procedure. Accuracy is expressed as the percent recovery of the spiked samples and repeatability (precision) as the RSD. The precision was assessed as the intra-day repeatability (RSDr) and inter-day reproducibility (RSD_R_). All experiments were carried out in quintuplicate on three different days. The intra-day precision was measured by comparing the standard deviation of the OTA recovery percentages of spiked samples acquired on the same day. The inter-day precision was determined by comparing the standard deviation of the OTA recovery percentages of the spiked samples on three separate days.

Blank (unspiked) samples were extracted from the matrices and analyzed to determine the specificity of the method and to identify interfering peaks that co-eluted with OTA. Identification of co-extracted compounds was assessed by monitoring the typical ion-chromatograms at the retention time intervals expected for OTA.

The linearity of the method was determined by quantifying the MS/MS peak area results for the standard solutions and the matrix-matched standard solutions in triplicate at six concentrations: 0.1, 1, 5, 10, 100, 500 µg kg^−1^. The linear regression equation for the standard solution was plotted, and its slope, intercept, and the correlation coefficient (R^2^) calculated.

The stability of OTA in the stock solvent solutions and in the matrices containing spiked samples (10 µg kg^−1^) was assessed monthly. All aforementioned tested samples were stored at −20 °C.

When using electrospray ionization, the presence of coextractives may have positive or negative effects on the chromatographic response of the analyte. And the matrix effect could be calculated as follows: matrix effect (ME%) = (slope of calibration curves in matrix − slope of calibration curves in solvent)/slope of calibration curves in solvent (×100%).
